# Supplementary material for: Quantifying and understanding the triboelectric series of inorganic non-metallic materials
Source: Nat Commun. 2020 Apr 29;11:2093. doi: 10.1038/s41467-020-15926-1 (PMC7190865; doi:10.1038/s41467-020-15926-1)
Supplement: Supplementary file 1 — Supplementary Information [file 41467_2020_15926_MOESM1_ESM.pdf]

Supplementary Information for

Quantifying and understanding the triboelectric series of inorganic non-metallic materials

by Haiyang et al.

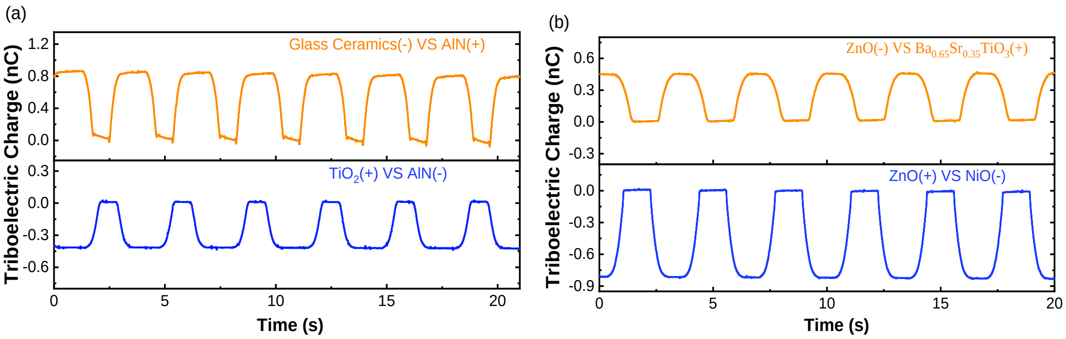

Supplementary Figure 1. The cross-check results charge transfer between various inorganic materials. (a) cross-check AlN with Glass ceramics and TiO_2, and (b) cross-check ZnO with $Ba_{0.65}Sr_{0.35}TiO_3$ and NiO.

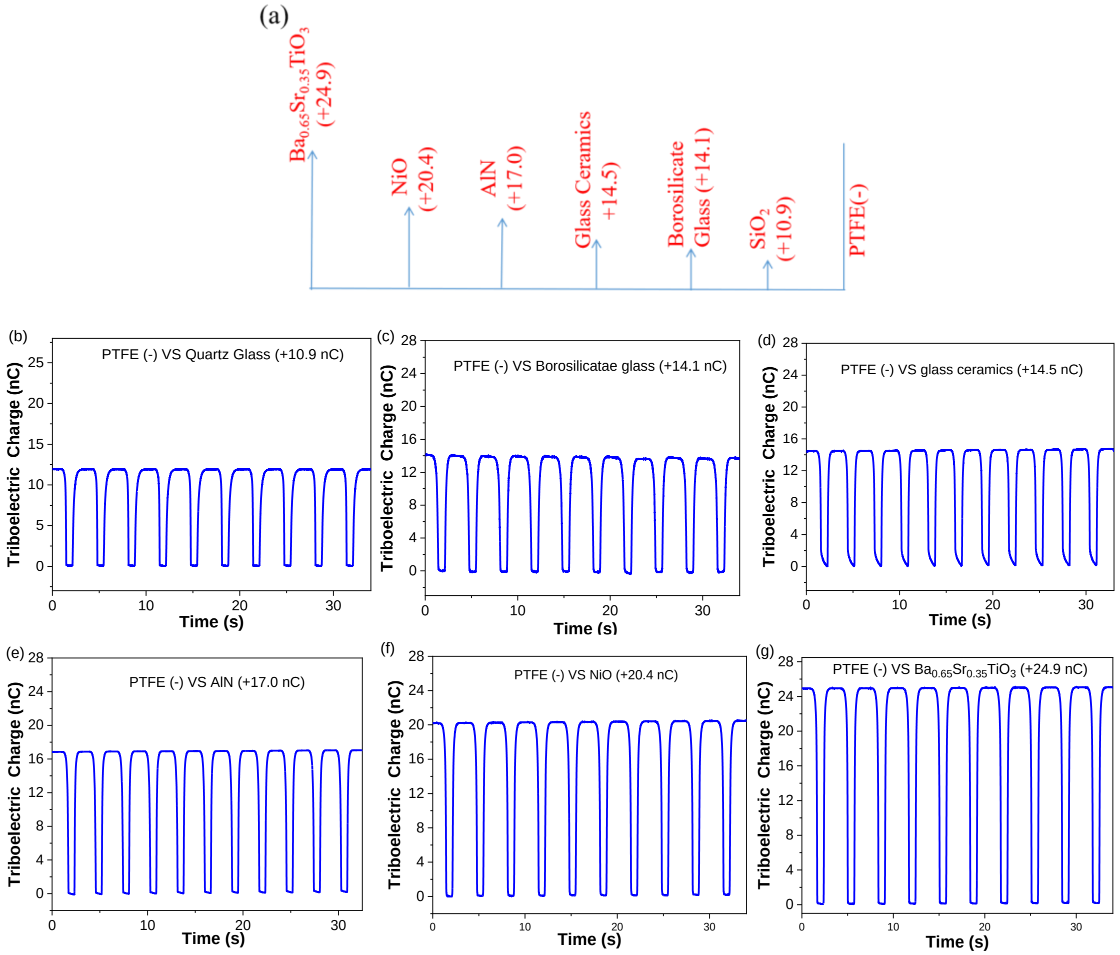

Supplementary Figure 2. The charge transfer between PTFE and various inorganic materials. (a) The ranking for the tested materials in the triboelectric series. (b-g) The measured charge transfer between PTFE and Quartz Glass (b), Borosilicate glass (c), High-Temperature Glass Ceramic (d), AlN (e), NiO (f), and $Ba_{0.65}Sr_{0.35}TiO_3$ (g).

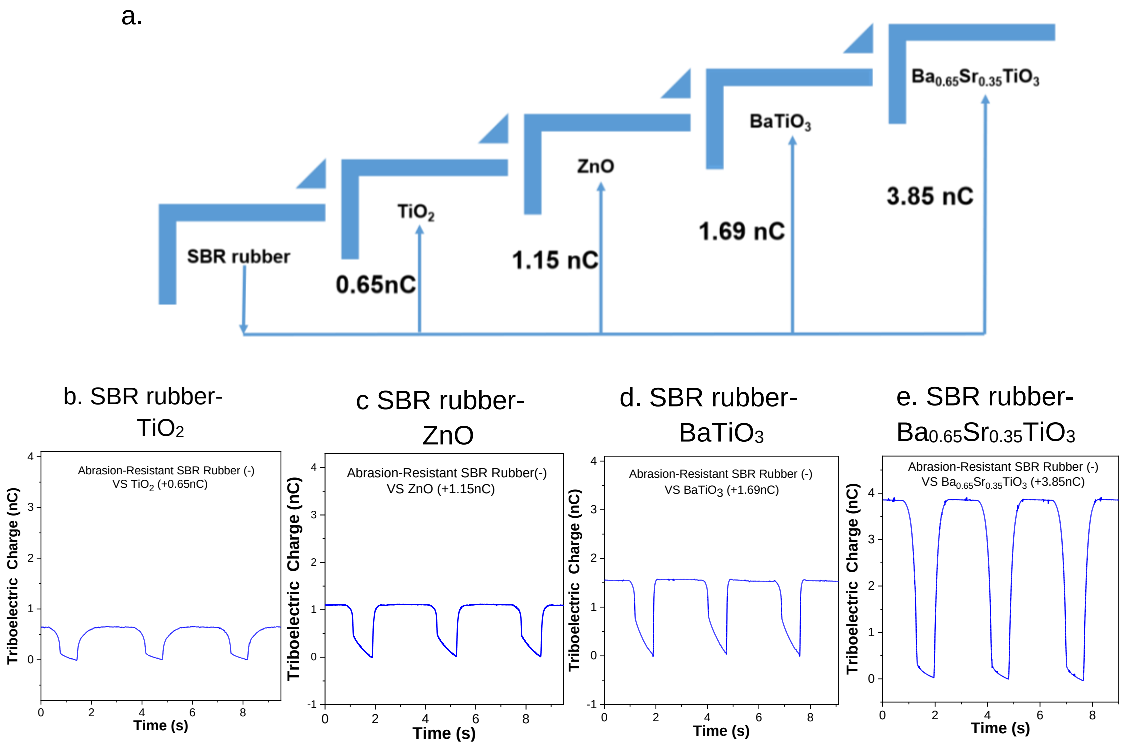

Supplementary Figure 3. The charge transfer between SBR rubber and various inorganic materials. (a) The ranking for the tested materials in the triboelectric series. (b-e) The measured charge transfer between SBR rubber and TiO_2 (b), ZnO (c), $BaTiO_3$ (d), and $Ba_{0.65}Sr_{0.35}TiO_3$ (e).

Supplementary Table 1. The details of tested materials. Note: mil is equal to one thousandth of an inch.

Materials	Vendor	Catalog No.	Synthesis Process	Thickness (mil)	Raw materials
Mica	Macmaster	8802K16			
Float glass	Beijing Kaifa Advanced Ceramics Technology Co., Ltd.			78.7	
Borosilicate glass	Macmaster	8476K12		125	
BeO	Guangzhou Beilong Electronics Co., Ltd.			27.6	
PZT-5	Zhejiang Gengjian Electronic Technology Co., Ltd.			78.7	
$MgSiO_3$	Synthesized		1、atmosphere：air 2、calcining temperature：1100℃ 3、calcining time：3h 4、sintering temperature：1400℃ 5、heating rate:3℃/min 6、sintering time：2h	63.3	MgO, SiO_2
$CaSiO_3$	Synthesized		1、atmosphere：air 2、calcining temperature：1100℃ 3、calcining time：3h 4、sintering temperature：1400℃ 5、heating rate:3℃/min 6、sintering time：2h	63.5	$CaCO_3$, SiO_2
$Bi_4Ti_3O_{12}$	Synthesized		1、atmosphere：air 2、calcining temperature：800℃ 3、calcining time：2h 4、sintering temperature：1200℃ 5、heating rate:3℃/min 6、sintering time：2h	61.9	Bi_2O_3,TiO_2
$Bi_{0.5}Na_{0.5}TiO_3$	Synthesized		1、atmosphere：air 2、calcining temperature：800℃ 3、calcining time：2h 4、sintering temperature：1160℃ 5、heating rate:3℃/min 6、sintering time：2h	62.4	Bi_2O_3,Na_2CO_3,TiO_2
$NiFe_2O_4$	Synthesized		1、atmosphere：air 2、calcining temperature：1100℃ 3、calcining time：6h 4、sintering temperature：1200℃ 5、heating rate:3℃/min 6、sintering time：6h	61.2	NiO,Fe_2O_3
$Ba_{0.65}Sr_{0.35}TiO_3$	Synthesized		1、atmosphere：air 2、calcining temperature：1100℃ 3、calcining time：3h 4、sintering temperature：1400℃ 5、heating rate:5℃/min 6、sintering time：3h	59.1	$BaCO_3$，$SrCO_3$,TiO_2

(To be continued)

(Continued)

Materials	Vendor	Catalog No.	Synthesis Process	Thickness (mil)	Raw materials
$BaTiO_3$	Synthesized		1、atmosphere：air 2、calcining temperature：1100℃ 3、calcining time：3h 4、sintering temperature：1350℃ 5、heating rate:5℃/min 6、sintering time：3h	59.1	$BaCO_3$,TiO_2
PZT-4	Zhejiang Gengjian Electronic Technology Co., Ltd.			100.4	
ZnO	Synthesized		1、atmosphere：air 2、sintering temperature：1200℃ 3、heating rate:5℃/min 4、sintering time：1.5h	60.3	ZnO
NiO	Synthesized		1、atmosphere：air 2、sintering temperature：1420℃ 3、heating rate:5℃/min 4、sintering time：3h	59.1	NiO
SnO_2	Synthesized		1、atmosphere：air 2、sintering temperature：1300℃ 3、heating rate:5℃/min 4、sintering time：2h	59.3	SnO_2
SiC	Guangzhou Beilong Electronics Co., Ltd.			78.7	
$CaTiO_3$	Synthesized		1、atmosphere：air 2、calcining temperature：1100℃ 3、calcining time：3h 4、sintering temperature：1350℃ 5、heating rate:5℃/min 6、sintering time：3h	61.2	$CaCO_3$ TiO_2
ZrO_2	Guangzhou Beilong Electronics Co., Ltd.			39.4	
Cr_2O_3	Synthesized		1、atmosphere：air 2、sintering temperature：1500℃ 3、heating rate:5℃/min 4、sintering time：3h	60.2	Cr_2O_3
Fe_2O_3	Synthesized		1、atmosphere：air 2、sintering temperature：800℃ 3、heating rate:5℃/min 4、sintering time：3h	59.6	Fe_2O_3
Al_2O_3	Fujian Huaqing Electronic Material Technology Co.,Ltd.		1、atmosphere：air 2、sintering temperature：1600℃ 3、heating rate:5℃/min 4、sintering time：3h	39.4	
TiO_2	Synthesized		1、atmosphere：air 2、sintering temperature：1350℃ 3、heating rate:5℃/min 4、sintering time：3h	60.3	TiO_2

(To be continued)

(Continued)

Materials	Vendor	Catalog No.	Synthesis Process	Thickness (mil)	Raw materials
AlN	Fujian Huaqing Electronic Material Technology Co.,Ltd.		1、atmosphere：N_2+H_2(5%)<br>2、sintering temperature：1800℃<br>3、heating rate:5℃/min 4、sintering time：3h	39.4	
BN	Guangzhou Beilong Electronics Co., Ltd.			78.7	
Clear Very High-Temperature Glass Ceramic	Macmaster	84815K41		197	
Ultra-High-Temperature Quartz Glass	Macmaster	1357T47		62.5	

Supplementary Table 2. The work functions and dielectric constants of tested materials

Materials	Work function (eV)	Dielectric constant
Float glass		4.7[1]
Borosilicate glass		4.5 - 6.2[1]
BeO	3.95-4.7[2]	7.15-7.55[1]
PZT-5	4.5[3]	1600-3400[1]
$MgSiO_3$		6.5[1]
$CaSiO_3$	4.35-4.48[4]	5-5.65[1]
$Bi_4Ti_3O_{12}$		112[1]
$Bi_{0.5}Na_{0.5}TiO_3$		400[1]
$NiFe_2O_4$		17[5]
$Ba_{0.65}Sr_{0.35}TiO_3$	4.5[3]	3688[1]
$BaTiO_3$	4.0-4.8[6]	2300[1]
PZT-4	3.9-4.6[7]	1550[1]
ZnO	4.1-5.3[8]	8.2-11[1]
NiO	4.4- 5.2[9]	11.9[1]
SnO_2	4.7[10]	8.5-16[1]
SiC	4.41-4.85[11]	9.7-10.03[1]
$CaTiO_3$	5.30-5.35[12]	165[1]

(To be continued)

(Continued)

Materials	Work function (eV)	Dielectric constant
ZrO_2	5.14[13]-5.55[14]	12.5[1]
Cr_2O_3	4.0[15]-4.8[16]	11.9-13.3[1]
Fe_2O_3	4.5-5.6[17]	12[1]
Al_2O_3	4.7-6[18]	9[1]
TiO_2	4.9-5.4[19]	86–170[1]
AlN	5.35[11]	8.7-9[1]
BN	B tip 5.843,N tip 7.203[20]	7.1[1]
Clear Very High-Temperature Glass Ceramic	—	—
Ultra-High-Temperature Quartz Glass	5.476-5.508[4]	4.41-4.6[1,4]
$CaTiO_3$	0.24	5.30-5.35[12]

Supplementary References

1 Haynes, W. M. Crc handbook of chemistry and physics : A ready-reference book of chemical and physical data 93rd edn12-124 (CRC Press, Boca Raton, 2012-2013).

2 Ahmadaghaei, N. & Noei, M. Density functional study on the sensing properties of nano-sized beo tube toward h2s. *J. Iran. Chem. Soc.* **11**, 725-731 (2014).

3 Quan, Z. *et al.* Interfacial characteristics and dielectric properties of ba0.65sr0.35tio3 thin films. *Thin Solid Films* **516**, 999-1005 (2008).

4 Manouchehri, H. R., Hanumantha Rao, K. & Forssberg, K. S. E. Triboelectric charge, electrophysical properties and electrical beneficiation potential of chemically treated feldspar, quartz and wollastonite. *Magn. Electr. Separ.* **11**, 9-32 (2002).

5 Lv, L. *et al.* Grain size effect on the dielectric and magnetic properties of nife2o4 ceramics. *Physica E Low Dimens. Syst. Nanostruct.* **43**, 1798-1803 (2011).

6 Schulmeyer, T. *et al.* Modification of batio3 thin films: Adjustment of the effective surface work function. *J. Mater. Chem.* **17**, 4563-4570 (2007).

7 Wang, C. Y. *et al.* Photocathodic behavior of ferroelectric pb(zr,ti)o-3 films decorated with silver nanoparticles. *Chem. Commun.* **49**, 3769-3771 (2013).

8 Gutmann, S., Conrad, M., Wolak, M. A., Beerbom, M. M. & Schlaf, R. Work function measurements on nano-crystalline zinc oxide surfaces. *J. Appl. Phys.* **111**, 123710 (2012).

9 Hietzschold, S. *et al.* Functionalized nickel oxide hole contact layers: Work function versus conductivity. *ACS Appl. Mater. Interfaces* **9**, 39821-39829 (2017).

10 Yu, S. H. *et al.* Optimization of sno2/ag/sno2 tri-layer films as transparent composite electrode with high figure of merit. *Thin Solid Films* **552**, 150-154 (2014).

11 Pelletier, J., Gervais, D. & Pomot, C. Application of wide-gap semiconductors to surface-ionization - work-functions of aln and sic single-crystals. *J. Appl. Phys.* **55**, 994-1002 (1984).

12 Lin, J. J. *et al.* In situ hydrothermal etching fabrication of catio3 on tio2 nanosheets with heterojunction effects to enhance co2 adsorption and photocatalytic reduction. *Catal. Sci. Technol.* **9**, 336-346 (2019).

13 Tsiplakides, D. & Vayenas, C. G. The absolute potential scale in solid state electrochemistry. *Solid State Ionics* **152**, 625-639 (2002).

14 Miyazaki, S., Narasaki, M., Ogasawara, M. & Hirose, M. Chemical and electronic structure of ultrathin zirconium oxide films on silicon as determined by photoelectron spectroscopy. *Solid State Electron.* **46**, 1679-1685 (2002).

15 Mejias, J. A., Staemmler, V. & Freund, H. J. Electronic states of the cr2o3(0001) surface from ab initio embedded cluster calculations. *J. Condens. Matter Phys.* **11**, 7881-7891 (1999).

16 Balouria, V. *et al.* Temperature dependent h2s and cl2 sensing selectivity of cr2o3 thin films. *Sens. Actuators, B Chem.* **157**, 466-472 (2011).

17 Chueh, Y. L., Lai, M. W., Liang, J. Q., Chou, L. J. & Wang, Z. L. Systematic study of the growth of aligned arrays of alpha-fe2o3 and fe3o4 nanowires by a vapor-solid process. *Adv. Funct. Mater.* **16**, 2243-2251 (2006).

18 Wang, Y., Li, Y. W., Yu, K. & Zhu, Z. Q. Controllable synthesis and field emission enhancement of al2o3 coated in2o3 core-shell nanostructures. *J. Phys. D Appl. Phys.* **44**, 105301 (2011).

19 Imanishi, A., Tsuji, E. & Nakato, Y. Dependence of the work function of tio2 (rutile) on crystal faces, studied by a scanning auger microprobe. *J. Phys. Chem. C* **111**, 2128-2132 (2007).

20 He, C. Y., Yu, Z. Z., Sun, L. Z. & Zhong, J. X. Work functions of boron nitride nanoribbons: First-principles study. *J. Comput. Theor. Nanosci.* **9**, 16-22 (2012).
